# Supplementary material for: Understanding resource driven female–female competition: ovary and liver size in sand gobies
Source: R Soc Open Sci. 2019 Sep 11;6(9):190886. doi: 10.1098/rsos.190886 (PMC6774974; doi:10.1098/rsos.190886)
Supplement: Abundance and biomass of benthic macrofauna [file rsos190886supp1.pdf]

Supplementary material I

Table S1

Sand gobies eat benthic macrofauna, primarily benthic infauna and small crustaceans [1, 2]. This table provides an overview of studies that have quantified abundance and biomass of soft-bottom macrofaunal communities, in or near areas representing our North Sea location (Bökevik Bay, by the Gullmar Fjord on the Swedish West Coast) and the Baltic Sea location (Tvärminne, by the Hanko Peninsula on the Finnish South Coast).

Reference	Figure / Table	Year	Location	Abundance (individuals/m^2)	Biomass (wet wt g/m^2)
Baltic Sea: Finnish South Coast					
[3]	Fig. 8	2000 - 2013	Archipelago Sea and Hanko Peninsula	-	100 - >300
[4]	Table 3	1994	Åland Archipelago	7872	197
[5]	Table 1	1993	Åland Archipelago and Hanko Peninsula	11392 (mean for localities A-D) and 12632 (locality E)	-
[6]	Table 3	2012 - 2014	Hanko Peninsula	67714 (mean for sites 1-21)	-
North Sea: Swedish West Coast					
[3]	Fig. 8	2000 - 2013	Northernmost part of Kattegat	-	50 - 300
[7]	Fig. 3	2003, 2004	Gullmars Fjord	approx. 4500	approx. 35
[8]	Fig. 2	2000	Bökevik	approx. 2000 (mean for weeks 1-8)	-

References

1. Aarnio, K, Sandberg, E, & Bonsdorff, E. 1991 Benthic predation on shallow-water macro- and meiofauna in the Baltic Sea: an experimental comparison between *Pomatoschistus minutus* (Pisces) and *Saduria entomon* (Crustacea). *Ann. Zool. Fennici* **28**, 41–48.

2. Zander CD, Hagemann T. 1986 Fluctuations of prey, abundance and biomass of gobies (Gobiidae, Pisces) in a shallow habitat of the western *Baltic Sea. Zool. Anz.* **216**, 289–304.

3. Gogina M, Nygård H, Blomqvist M, Daunys D, Josefson AB, Kotta J, et al. 2016. The Baltic Sea scale inventory of benthic faunal communities. *ICES J. Mar. Sci.* **73**, 1196–1213. (doi:10.1093/icesjms/fsv265)

4. Bonsdorff E, Diaz RJ, Rosenberg R, Norkko A, Cutter, GR. 1996 Characterization of soft-bottom benthic habitats of the Åland Islands, northern Baltic Sea. *Mar. Ecol. Prog. Ser.* **142**, 235–245.

5. Boström C, Bonsdorff E. 1997 Community structure and spatial variation of benthic invertebrates associated with *Zostera marina* (L.) beds in the northern Baltic Sea. *J. Sea Res.* **37**, 153–166. (doi: 10.1016/S1385-1101(96)00007-X)

6. Jokinen H, Wennhage H, Ollus V, Aro E, Norkko A. 2016 Juvenile flatfish in the northern Baltic Sea - long-term decline and potential links to habitat characteristics. *J. Sea Res.* **107**, 67–75. (doi:10.1016/j.seares.2015.06.002)

7. Stål J, Pihl L, Wennhage, H. 2007 Food utilisation by coastal fish assemblages in rocky and soft bottoms on the Swedish west coast: Inference for identification of essential fish habitats. *Estuar. Coast. Shelf Sci.* **71**, 593–607. (doi:10.1016/j.ecss.2006.09.008)

8. Jackson AC, Rundle SD. 2008 Diet–shifts by an estuarine goby (*Pomatoschistus microps*) in the face of variable prey availability. *J. Exp. Mar. Biol. Ecol.* **361**, 1–7. (doi:10.1016/j.jembe.2008.03.007)
