# Supplementary material for: Understanding resource driven female–female competition: ovary and liver size in sand gobies
Source: R Soc Open Sci. 2019 Sep 11;6(9):190886. doi: 10.1098/rsos.190886 (PMC6774974; doi:10.1098/rsos.190886)
Supplement: Reproductive Maturity Index (RMI) [file rsos190886supp2.pdf]

Supplementary Material II

**RMI (Reproductive Maturity Index)**		
0.75	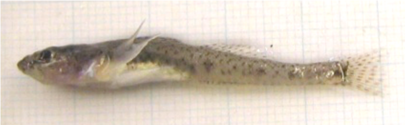	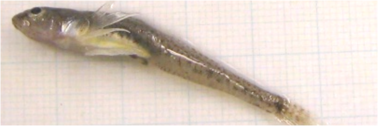
1.00	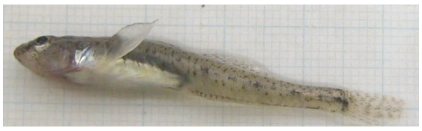	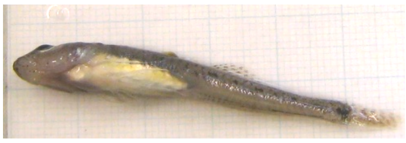
1.25	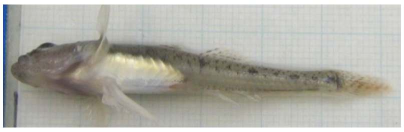	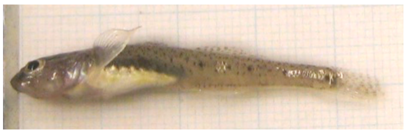
1.50	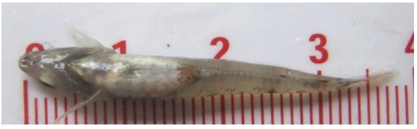	
1.75	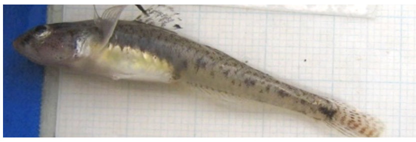	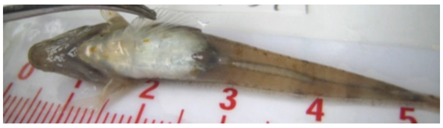
2.00	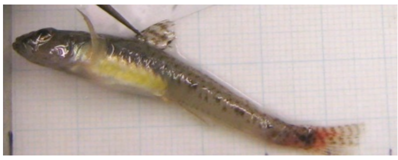	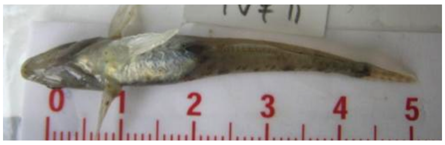
2.25	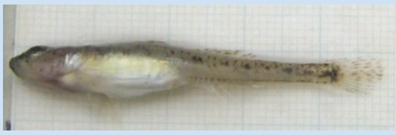	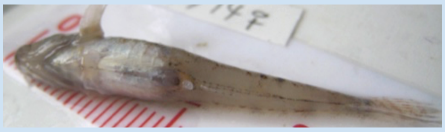
2.50	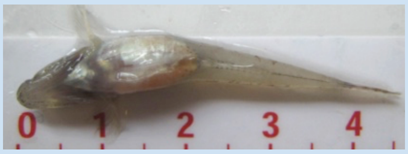	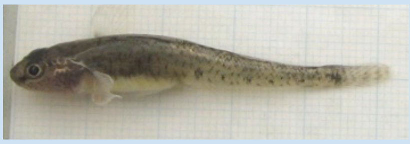
2.75	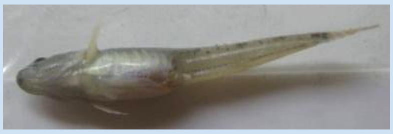	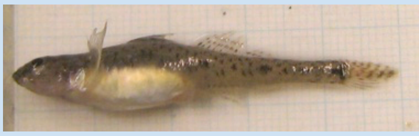
3.00	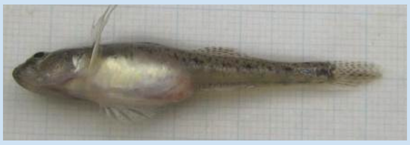	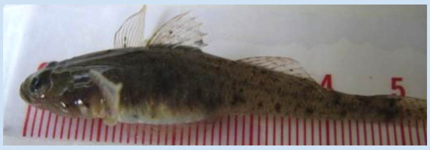

Figure S1. Reproductive maturity index (RMI) of sand goby (*Pomatoschistus minutus*) females, ranging 0.75-3.00. Photographed females were either caught in June 2014, in Bökevik, Sweden, or Tvärminne, Finland. RMI values ≥ 2.25 indicate females that are ready to spawn.
